# From Exposure to Psychological Harm: A Socioecological and Trauma-Informed Narrative Review of Bullying, School Violence, and Youth Mental Health

**DOI:** 10.3390/bs16101815

**Published:** 2026-10-04

**Authors:** Ghada Shahrour, Fatma M. Ibrahim

**Affiliations:** 1 Faculty of Nursing, Jordan University of Science and Technology, Irbid P.O. Box 11172, Jordan; 2 RAK College of Nursing, RAK Medical and Health Sciences University, Ras Al Khaimah P.O. Box 11172, United Arab Emirates; 3 Faculty of Nursing, Mansoura University, Mansoura 35516, Egypt

**Keywords:** bullying, school violence, cyberbullying, peer victimization, adolescent mental health, school climate, trauma-informed care, prevention, implementation science

## Abstract

Bullying and school violence are preventable exposures and potentially modifiable risk factors for adverse youth mental health and educational outcomes. This critical narrative review integrates epidemiological, longitudinal, genetically informed, quasi-experimental, meta-analytic, and intervention evidence on bullying, cyberbullying, selected forms of school violence, and their psychological sequelae. The evidence base available at the original submission was reassessed during revision through a complete structured rerun of PubMed/MEDLINE, APA PsycINFO, ERIC, Scopus, Web of Science Core Collection, Google Scholar, major publisher platforms, and authoritative organizational sources from 15 to 27 August 2026, followed by a final update on 8 September 2026; reporting was guided by the Scale for the Assessment of Narrative Review Articles (SANRA). Evidence supports robust associations with depression, anxiety, trauma symptoms, loneliness, sleep disturbance, self-harm, suicidality, substance use, and externalizing difficulties, although effect estimates differ in design, metric, and certainty. We propose an evidence-informed, not prospectively validated, mechanism-to-action framework. Within the reviewed corpus, direct prospective mediation evidence was identified for interpersonal distrust and school belonging; longitudinal evidence supports a rumination-sleep sequence; and threat appraisal, humiliation/shame, and social defeat remain theory-supported candidate mechanisms. Socioecological theory organizes influences across individual, peer, school, family, community, policy, and digital levels. Trauma-informed principles are used as an interpretive and ethical lens; evidence for whole-school trauma-informed models remains limited. Whole-school anti-bullying programs modestly reduce exposure, but average effects on internalizing symptoms are small and heterogeneous. Systems should therefore coordinate exposure prevention with assessment, treatment, and follow-up of psychological harm. Progress should be judged by safety, equity, belonging, functioning, and recovery, not incident counts alone.

## 1. Introduction

Schools are expected to provide safety, belonging, and developmental opportunity. For many students, however, the school ecology also contains repeated peer aggression, identity-based harassment, physical or sexual violence, threats, weapon-related fear, and digitally mediated abuse that moves continuously between school and home. UNESCO (2019) estimated that 32% of students had been bullied by peers at least once in the preceding month and that a similar proportion had experienced physical violence. More recent Health Behaviour in School-aged Children data from 44 countries and regions found that approximately 11% of adolescents reported school bullying victimization and 15% reported cyberbullying victimization; cybervictimization increased between 2018 and 2022 (Biswas et al., 2020; Cosma et al., 2024). In the 2023 United States Youth Risk Behavior Survey, bullying victimization clustered with frequent social-media use, persistent sadness or hopelessness, and suicide risk (Young et al., 2024). Although prevalence estimates vary with definitions, reference periods, sampling, age, and cultural norms, the convergence of international evidence establishes bullying and school violence as population-level threats rather than isolated disciplinary problems.

Bullying is conventionally defined as intentional aggression that is repeated, or likely to be repeated, within a relationship marked by power imbalance (Olweus, 2013). Peer victimization is a broader descriptive term for harmful treatment by peers and may include episodes that do not satisfy all bullying criteria. Cyberbullying retains harm and power asymmetry but complicates repetition because a single post can be copied, commented upon, and repeatedly viewed; anonymity, technological expertise, audience size, and permanence may create power (Smith et al., 2008; Zhu et al., 2021). School violence is broader still and includes physical attacks, fighting, sexual harassment or coercion, psychological aggression, threats, weapon-related incidents, staff-to-student violence, and witnessing. Table 1 is the conceptual anchor for these nested but non-equivalent constructs. Conflation obscures bullying-specific dynamics; excessive narrowing excludes acute, institutional, and digital harms relevant to school safety and mental health.

The mental health literature is correspondingly fragmented. Traditional bullying, cyberbullying, physical violence, school-shooting exposure, and bias-based harassment are frequently reviewed in separate silos. Studies also divide students into victims, perpetrators, perpetrator–victims, and bystanders, although these roles can change across time and contexts. Moreover, an observed association is not automatically causal: pre-existing anxiety, impulsivity, social-communication difficulties, family adversity, and genetic liability may increase both exposure and later symptoms. Strong inference therefore requires triangulation across prospective cohorts, genetically informed and quasi-experimental designs, dose–response patterns, and intervention studies.

Previous syntheses have documented adverse outcomes (Arseneault, 2018; Han & Yang, 2025; Moore et al., 2017), reciprocal longitudinal associations (Christina et al., 2021; Reijntjes et al., 2010), school-violence consequences (Polanin et al., 2020), and particular modalities such as cyberbullying. The distinctive contribution of this review is narrower and integrative: it maps candidate psychological mechanisms onto observable indicators, evaluation targets, and role-specific actions while separating reduction in exposure from recovery after harm. The resulting two-track architecture is compatible with established multi-tiered systems of support rather than a replacement for them (McIntosh & Goodman, 2016). Its added value is the explicit mechanism-to-measurement bridge across socioecological levels; the complete architecture has not been prospectively tested as a package.

## 2. Materials and Methods

### 2.1. Review Design and Aim

A critical narrative-review design was selected because the objective was explanatory integration across heterogeneous exposures, outcomes, disciplines, and study designs rather than estimation of a single pooled effect. The review was planned and reported with reference to SANRA (Baethge et al., 2019). No protocol was registered because the work was not designed as a systematic review; accordingly, the methods below support transparency and auditability but should not be interpreted as PRISMA-compliant exhaustive screening.

The review aimed to: (1) clarify the conceptual boundaries between bullying and other school violence; (2) synthesize evidence on mental health outcomes and causal direction; (3) identify mechanisms, moderators, and protective processes; (4) critically evaluate intervention evidence; and (5) propose a mechanism-to-action framework and research agenda relevant to educators, health professionals, families, digital platforms, and policymakers.

### 2.2. Search Strategy and Evidence Selection

The original submission on 29 July 2026 was based on literature identified before submission. During revision, both authors conducted a complete structured rerun of the purposive searches from 15 to 27 August 2026 and a final update on 8 September 2026. The sources were PubMed/MEDLINE; APA PsycINFO and ERIC (EBSCOhost); Scopus; Web of Science Core Collection; Google Scholar for citation discovery; ScienceDirect, SpringerLink, Wiley Online Library, Taylor & Francis Online, SAGE Journals, and JAMA Network for targeted full-text and citation verification; and reports or surveillance pages from UNESCO, WHO, CDC, and the Community Preventive Services Task Force. Database-specific fields, limits, dates, and representative full strings are provided in Supplementary File S1. Search concepts covered exposure, school-aged populations, mental health and educational outcomes, candidate mechanisms, moderators, and prevention or treatment. Searches emphasized English-language peer-reviewed work published from 2000 through 8 September 2026; age filters were not imposed electronically because inconsistent indexing could reduce sensitivity.

Eligible sources primarily concerned school-aged children or adolescents and bullying, peer victimization, cyberbullying, or violence occurring in school-related social systems. University-only, workplace, sibling, dating-partner, and unrelated family/community violence studies were excluded unless they contributed uniquely relevant theory or mechanism evidence. Systematic reviews/meta-analyses, representative surveillance, prospective cohorts, within-family/genetically informed and quasi-experimental studies, randomized trials, and direct mediator/moderator tests were prioritized over cross-sectional studies and opinion pieces. When multiple reports addressed the same question, recency, methodological rigor, sample breadth, directness, precision, and cross-context replication guided selection. Backward searching examined reference lists of included syntheses and landmark studies; forward searching used citation indexes and Google Scholar. Because the search was iterative and purposive, a defensible denominator of all screened records cannot be reconstructed and no PRISMA flow count is claimed; the cited corpus is auditable through the reference list and Supplementary Table S1.

Evidence extraction used a structured chart covering design, population, setting, exposure, outcome, effect estimate, uncertainty, temporality, confounding control, principal limitation, and relevance to the framework. Both authors independently verified eligibility and extraction for quantitative anchor studies and resolved differences through discussion and consensus. Other retained sources were checked by the second author for directness and interpretive accuracy. Evidence was synthesized by conceptual matrix rather than pooled: findings were grouped as (i) empirically demonstrated associations or intervention effects, (ii) evidence-informed implications supported indirectly or across adjacent studies, and (iii) author-proposed hypotheses or practice recommendations. This hierarchy is shown in Table 2, Table 3 and Table 4 and Supplementary File S1.

### 2.3. Derivation and Status of the Conceptual Framework

The framework was developed iteratively during synthesis rather than specified a priori. Deductive domains came from socioecological theory (Bronfenbrenner, 1979; Espelage & Swearer, 2004), developmental psychopathology, Social Safety Theory (Slavich, 2020), trauma-informed principles (Substance Abuse and Mental Health Services Administration [SAMHSA], 2014), and prevention science. Inductive refinement occurred when recurrent mediators, moderators, and intervention targets were charted across the retained literature. Candidate pathways were retained when they had direct prospective mediation evidence, a replicated temporal association, or a clearly specified theoretical rationale with observable indicators. Alternative explanations including stable vulnerability, social selection, common-method bias, family adversity, genetic liability, and reverse direction were explicitly retained rather than absorbed into a single causal chain.

**Figure 1:**
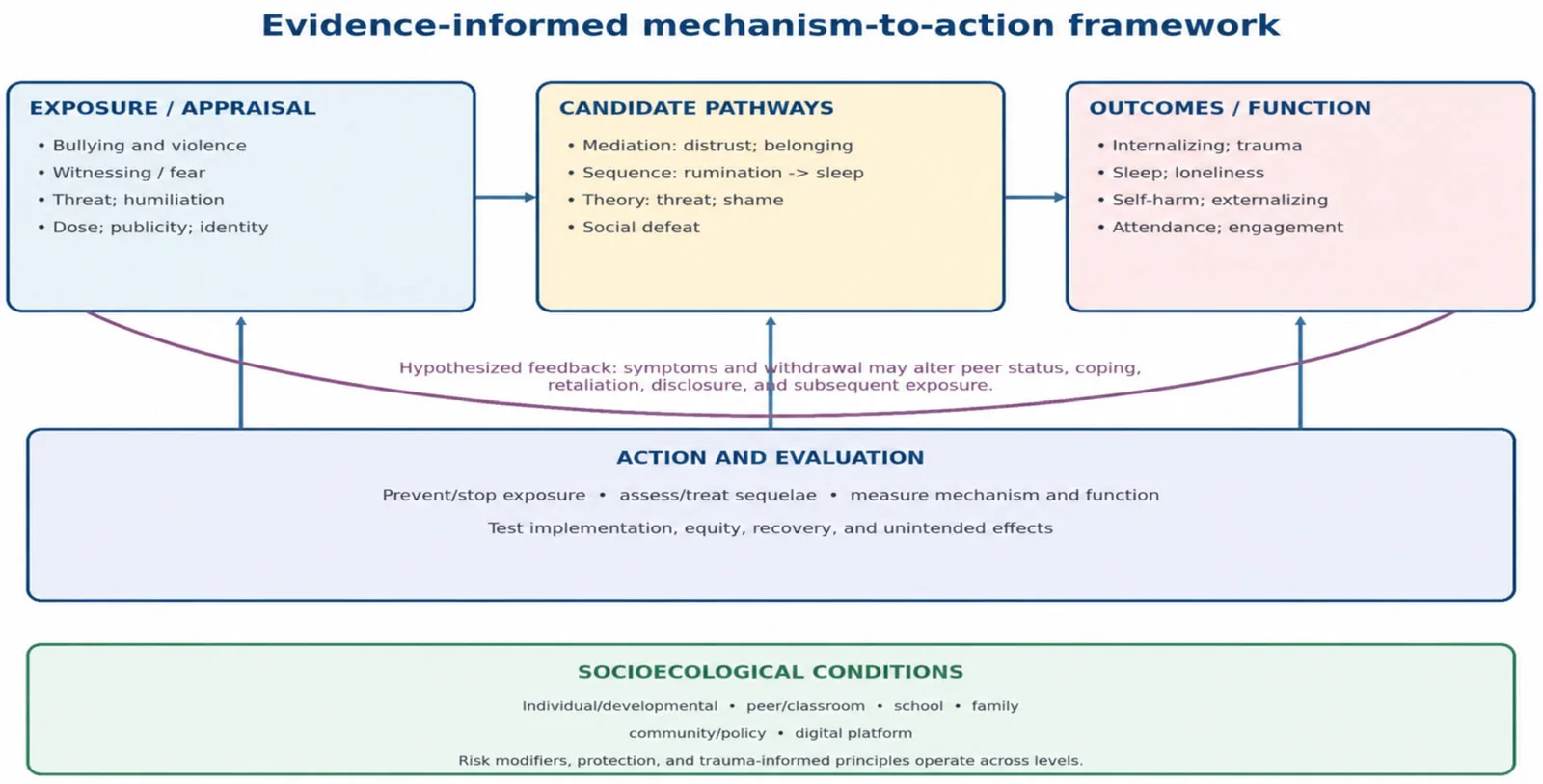
Proposed evidence-informed socioecological mechanism-to-action framework. Solid arrows indicate the principal hypothesized sequence; the return loop represents possible reciprocal vulnerability, social selection, revictimization, school withdrawal, rumination/sleep effects, retaliation, and peer-status change. The lower band indicates multilevel conditions that may modify exposure, pathways, outcomes, and intervention response. The framework is hypothesis-generating and has not been validated as a complete causal model.

Intervention components were mapped to mechanisms only when their proposed function plausibly altered the exposure, appraisal, mediator, or recovery outcome; mapping did not count as proof of mediated intervention effect. Figure 1 and Table 3 and Table 4 therefore constitute a proposed evidence-informed conceptual framework and hypothesis-generating logic model, not a validated causal model or a fully tested integrated intervention. The framework would be challenged if prospective within-person or intervention-mediated analyses consistently showed that changes in the proposed mechanisms did not precede or explain changes in symptoms or functioning, or if equally rigorous studies supported competing pathways more strongly.

## 3. Results: Critical Narrative Synthesis

### 3.1. Prevalence Is Substantial, but Measurement Shapes the Burden

Global burden estimates are not interchangeable. Definition-based measures first present repetition, intent, and power imbalance and then ask respondents whether they were bullied; behavior-based measures ask about concrete acts without requiring the label. Single global items are efficient but cannot characterize modality or dose, whereas multi-item instruments improve content coverage yet differ substantially in included behaviors. Reviews have found wide variation in terminology and operationalization and that few measures capture all defining elements (Vivolo-Kantor et al., 2014). Thresholds for “repeated” also vary—from two or three times within a term to weekly exposure changing prevalence estimates. Informant matters: self-, peer-, teacher-, and administrative reports identify overlapping but different groups, with covert relational, sexual, and cyber aggression especially likely to be absent from adult records (Cornell & Brockenbrough, 2004).

Cross-national comparisons additionally assume that items function equivalently across languages, ages, gender groups, and educational systems. Translation, willingness to use the label “bullying,” digital access, and culturally patterned interpretations of power can produce differential item functioning; measurement invariance is seldom tested before prevalence ranks are compared. Reported cybervictimization ranges in global reviews span from single digits to more than 50% depending on definition, reference period, platform, and sampling frame (Zhu et al., 2021). Accordingly, HBSC, GSHS, and national estimates should be interpreted as instrument- and context-specific rather than interchangeable global constants. The clinically important observation is that combined offline and online victimization is associated with greater psychopathology than either modality alone (Li et al., 2024).

Broader school violence is even more heterogeneous. A single physical assault, chronic threat, sexual victimization, repeated fighting, and exposure to a fatal school shooting should not be collapsed into a single undifferentiated variable. At the same time, narrow classification can conceal cumulative burden. A student who witnesses fights, fears a weapon incident, experiences bias-based harassment, and is excluded online may not meet a strict threshold for any one exposure yet may live in a sustained state of social unsafety. Measurement should therefore capture modality, frequency, duration, severity, audience, role, context, identity-based content, perceived power, disclosure, response, and overlap with family or community violence.

### 3.2. Mental Health Outcomes and the Strength of Causal Inference

The association between victimization and internalizing symptoms is highly replicated, but the magnitude depends on design and metric. Ye et al. (2023) synthesized 31 cross-sectional studies (*n* = 133,688): pooled odds ratios for depressive symptoms were 2.77 (95% CI 2.29–3.35) for victims, 1.73 (1.34–2.23) for perpetrators, and 3.19 (2.54–4.01) for perpetrator–victims. Heterogeneity remained high (I^2^ > 50%) and publication bias was detected. These odds ratios compare odds, not risks; because depressive symptoms are not rare, they should not be described as equivalent proportional increases in probability or as near tripling of absolute risk.

Suicidality requires precise interpretation. Across 34 studies (66 effect sizes; *n* = 284,375), peer victimization was associated with suicidal ideation (random-effects OR 2.23, 95% CI 2.10–2.37; I^2^ = 85.2%); across nine studies (13 effect sizes; *n* = 70,102), it was associated with suicide attempts (OR 2.55, 1.95–3.34; I^2^ = 88.9%) (van Geel et al., 2014). High heterogeneity and predominantly cross-sectional self-report limit causal interpretation. Bullying is therefore a clinically relevant risk marker and potentially modifiable contributor that warrants direct, confidential assessment, not a monocausal explanation for any individual suicidal outcome.

Prospective evidence strengthens temporal ordering but requires attention to level of analysis. Conventional cross-lagged meta-analyses indicate that youth who are more victimized than peers tend later to report more internalizing symptoms and that youth with more symptoms tend later to report more victimization (Christina et al., 2021; Reijntjes et al., 2010). These between-person rank-order associations do not by themselves demonstrate a self-reinforcing process within the same young person. Random-intercept cross-lagged models, intensive longitudinal studies, and intervention-mediated analyses are needed to separate stable between-person differences from within-person change. The framework therefore represents reciprocal vulnerability and revictimization as testable possibilities, not established individual-level feedback effects.

Genetically informed and quasi-experimental studies provide more stringent tests. Twin-difference analyses support direct contributions to several concurrent outcomes, but many longer-term estimates attenuate after accounting for shared familial and genetic liabilities (Silberg et al., 2016; Singham et al., 2017). Singham et al. (2017) reported that most adjusted effects attenuated by five years, while associations with paranoid thoughts and cognitive disorganization persisted. A meta-analysis of quasi-experimental studies likewise found adverse short- and long-term consequences smaller than conventional observational estimates (Schoeler et al., 2018). The defensible conclusion is that bullying probably contributes causally to some outcomes and periods while reciprocal vulnerability, social selection, and residual confounding remain material.

Long-term follow-up indicates that severe or chronic childhood bullying can predict adult depression, anxiety disorders, suicidality, poorer social relationships, and reduced socioeconomic functioning (Copeland et al., 2013; Wolke & Lereya, 2015). Persistence is not inevitable. Attenuation over time in stringent designs is evidence of potential recovery, particularly when exposure stops and protective relationships, effective coping, and treatment become available (Martínez et al., 2024). This distinction matters ethically: risk estimates should motivate early action without constructing victimization as a permanent identity or fixed prognosis.

### 3.3. Cyberbullying Adds Persistence, Audience, and Loss of Control

Cyberbullying is psychologically consequential not simply because it uses technology, but because digital affordances alter the experience of power. Anonymity can make threat origin uncertain; rapid sharing expands the audience; searchable and replicable content can persist; quantitative feedback such as likes or shares publicly signals peer endorsement; and continuous connectivity reduces opportunities for recovery. Qualitative syntheses show that young people evaluate cyberbullying through perceived intent, publicity, repetition, controllability, and the adequacy of adult response, not merely through platform or message type (Dennehy et al., 2020).

Meta-analytic and systematic-review evidence links cybervictimization with internalizing symptoms, externalizing problems, self-harm, and suicidality (John et al., 2018; Kowalski et al., 2014), and a longitudinal synthesis of 27 studies (*n* = 13,497) reported a pooled prospective correlation of r = 0.23 (Lee et al., 2026). This is a pooled zero-order association across heterogeneous lags and adjustment strategies, not a within-person causal estimate. Longitudinal evidence indicates that depression, anxiety, negative self-cognitions, and substance-related difficulties can both precede and follow cyberbullying involvement (Fahy et al., 2016; Marciano et al., 2020), while repeated cyber and traditional victimization may also predict later post-traumatic stress symptoms (Holfeld & Mishna, 2021). In the meta-analysis by van Geel et al. (2014), cyberbullying had a larger association with suicidal ideation (OR 3.12, 95% CI 2.40–4.05; three studies) than traditional bullying (OR 2.16, 2.05–2.28), but the small cyberbullying evidence base requires caution. Rumination is plausible in digital contexts because content can be revisited and anticipated; longitudinal evidence links cybervictimization to rumination and subsequent sleep problems (Jose & Vierling, 2018).

Online and offline interventions should therefore be coordinated. Telling a student to disconnect may remove social support, learning access, and normative peer participation while leaving harmful material intact. Effective response requires evidence preservation, platform reporting, privacy protection, peer-network management, school action when the conflict affects learning or safety, and mental health assessment proportional to impact rather than to whether the incident occurred on school grounds.

### 3.4. A Mechanism-to-Action Account of Psychological Harm

The exposure-outcome association is best represented as a probabilistic, reciprocal system rather than a uniform cascade. Social Safety Theory proposes that sustained threats to social connection can influence health through psychological, neural, immunological, and behavioral pathways (Slavich, 2020). In 10,000 participants from the UK Millennium Cohort Study, bullying at age 11 predicted greater interpersonal distrust at age 14, which partially mediated mental health difficulties at age 17 (Tsomokos & Slavich, 2024). This provides prospective mediation support for distrust, although it does not validate every pathway in the proposed framework.

Evidence status differs across candidate mechanisms. Interpersonal distrust has direct prospective mediation support (Tsomokos & Slavich, 2024); school belonging has longitudinal mediation evidence (Arslan & Allen, 2021); and rumination followed by sleep disruption has temporal support (Jose & Vierling, 2018; Sutter et al., 2023). By contrast, threat appraisal, humiliation/shame, and social defeat are theoretically coherent and clinically observable but have not yet been established as prospective mediators of the victimization-to-symptom association. They are therefore labeled candidate pathways. This distinction prevents theoretical plausibility from being mistaken for demonstrated mediation.

School disconnection is a plausible institutional pathway. Victimization can reduce acceptance, respect, and perceived safety and thereby accompany absenteeism, concentration problems, disengagement, and reduced access to supportive relationships. Longitudinal evidence indicates that school belonging can mediate associations between victimization, emotional problems, and well-being (Arslan & Allen, 2021). The healthy context paradox—poorer adjustment among students who remain victimized in otherwise low-victimization settings—has between-person and intervention-based support (Huitsing et al., 2019), but evidence separating within-person change from stable differences remains limited. It should be treated as a contextual hypothesis requiring multilevel within-person tests, not a universal effect.

Witnesses are not psychologically neutral. Observing repeated bullying may generate fear of becoming the next target, helplessness, guilt for not intervening, moral distress, or normalization of aggression. Bystander status has been associated with anxiety, depressive symptoms, and broader behavioral or mental health burden (Evans et al., 2019; Midgett & Doumas, 2019; Rivers et al., 2009). At the same time, peers are powerful intervention agents: defending behavior can disrupt social rewards for perpetrators and affirm the target’s belonging. Bystander interventions should build safe, developmentally appropriate options seeking adult help, documenting, checking in privately, interrupting when safe without transferring responsibility for safety from adults to children.

Perpetration also requires a mental health and developmental lens without excusing harm. Some perpetrators gain social status and may show few immediate internalizing symptoms; others display impulsivity, conduct problems, trauma exposure, family violence, or hostile norms. Perpetrator–victims typically show the most complex profile because they combine exposure-related distress with aggression and disciplinary risk (Holt et al., 2015; Ye et al., 2023). Purely punitive responses can increase exclusion and conceal underlying needs. Proportionate accountability should be paired with functional assessment, skills development, family engagement, and monitoring for retaliation.

### 3.5. Who Is Most Vulnerable—And Why

Risk is patterned by exposure, social power, and access to protection rather than by a single demographic variable. Chronicity, severity, multiple perpetrators, public dissemination, overlap across online and offline settings, and combined victim-perpetrator involvement consistently mark higher risk. Prior depression, anxiety, attention difficulties, social-communication differences, or trauma may intensify threat appraisal and impede help-seeking, while victimization can worsen the same vulnerabilities. Assessment should therefore distinguish pre-existing need from victimization-attributable change without treating either as evidence that the target caused the abuse.

Children and adolescents with neurodevelopmental or psychiatric conditions have substantially higher odds of traditional and cyber victimization, perpetration, and perpetrator–victim involvement than comparison groups. A 2024 systematic review and meta-analysis reported particularly elevated odds for traditional victimization (OR 2.85) and perpetrator–victim status (OR 3.66), with bullying involvement adding to internalizing and externalizing burden (Abregú-Crespo et al., 2024). Communication differences, behavioral visibility, dependence on adults, segregated settings, and fewer reciprocal friendships can increase exposure and reduce disclosure. Accessible reporting, disability-informed safeguarding, and individualized communication supports are essential.

LGBTQ+ youth experience both general and bias-based bullying, with strong links to depression, suicidality, and other internalizing outcomes (Gower et al., 2018). A meta-analysis of LGBTQ+ young people with self-harm or suicide experiences found high prevalence of victimization and mental health difficulty and greater odds than among cisgender heterosexual counterparts with similar outcomes (Williams et al., 2021). Minority stress explains why identity-based harassment can do more than add another incident: it signals devaluation of the social group, may be reinforced by institutional policy or silence, and can threaten disclosure, family support, and access to affirming care (Meyer, 2003). Protective school strategies must explicitly address sexual orientation, gender identity and expression, and confidentiality rather than relying solely on generic civility language. Student alliances or comparable affirming groups are associated with lower victimization, although evidence is mostly observational (Marx & Kettrey, 2016).

Racial, ethnic, religious, citizenship, and migrant status can shape both exposure and meaning. Average prevalence comparisons across broad categories are inconsistent, but bias-based bullying is associated with more severe health consequences than non-bias-based bullying, and cultural identity can be protective when supported (Xu et al., 2020). Interventions that ignore prejudice may misclassify targeted harassment as interpersonal conflict and shift responsibility onto the victim. Equity-sensitive systems should record motive, examine disciplinary disproportionality, provide culturally and linguistically accessible reporting, and include affected communities in prevention design.

Gender comparisons should avoid simplistic conclusions. Boys more often report physical fighting and some forms of perpetration; girls may report more relational or cybervictimization in particular contexts and often show higher internalizing symptoms after interpersonal stress. Gender-diverse youth face distinct identity-based risks. Variation within gender groups is larger than many average differences, and measurement, disclosure, culture, and platform use modify observed patterns (Cosma et al., 2024; Lee et al., 2026). Developmental stage likewise changes exposure, interpretation, autonomy, and available support. Prevention should be developmentally sequenced rather than uniform across primary and secondary school.

### 3.6. Broader School Violence: Acute Trauma, Chronic Fear, and Institutional Trust

Bullying occurs within a larger safety ecology, but evidence should not be generalized across violence forms. Physical assault, serious fighting, threats, weapon exposure, and witnessing can produce acute injury, fear, and trauma symptoms without repetition or power imbalance; longitudinal synthesis links school-violence involvement with later mental health, academic, and delinquency outcomes (Polanin et al., 2020). Peer sexual harassment and coercion additionally involve gendered power, stigma, and safeguarding concerns; longitudinal evidence associates sexual-harassment victimization with subsequent distress, substance use, and further victimization (Chiodo et al., 2009). These exposures require distinct measurement and response pathways rather than classification as bullying by default.

School shootings represent an extreme, context-specific form of violence, most heavily studied in the United States. Concern, worry, and stress about school violence or shootings prospectively predicted anxiety and panic symptoms in adolescents, with some racial/ethnic variation in depressive associations (Riehm et al., 2021). In a quasi-experimental analysis of 44 fatal school shootings, local exposure was associated with a 21.4% increase in youth antidepressant use during the following two years (Rossin-Slater et al., 2020). A broader systematic review of interpersonal gun-violence exposure found associations with post-traumatic stress, depressive symptoms, substance use, disruptive behavior, and gun carrying, with greater harm among those closer to the event (Abba-Aji et al., 2024). These findings show that direct injury is not the only relevant endpoint; fear, grief, media exposure, disrupted routines, and collective trauma also require planned response.

Staff-to-student violence also warrants explicit attention. Corporal punishment and humiliating discipline remain legal or socially tolerated in some settings and are associated with adverse behavioral, mental health, and educational outcomes (Gershoff, 2017). Because disclosure and administrative recording are shaped by authority gradients, studies limited to peer perpetrators underestimate the full school safety ecology. International recommendations must therefore be adapted to legal protections, staffing, reporting infrastructure, language, and access to child and adolescent mental health services.

Safety measures can protect or inadvertently harm. A national social-media study found that active-shooter drills were followed by increases in anxiety, stress, and depressive-language indicators among students, parents, and teachers, although the observational design cannot establish clinical incidence (ElSherief et al., 2021). This U.S.-specific evidence should not be generalized to routine emergency preparedness or to all countries. Highly realistic drills, conspicuous surveillance, and exclusionary discipline should therefore be evaluated for perceived danger, differential burden, and unintended suppression of disclosure. This recommendation is evidence-informed; specific local protocols require contextual risk assessment and should not substitute for credible prevention, fair procedures, and accessible psychosocial support.

### 3.7. What Intervention Evidence Actually Shows

Bullying is a group and organizational process, not merely a dyadic conflict. Bronfenbrenner’s ecological systems theory (Bronfenbrenner, 1979) and bullying-specific socioecological applications (Espelage & Swearer, 2004; Swearer et al., 2010) locate risk and protection across the individual, peer group, classroom, school, family, community, policy, and historical context. Participant-role research shows that assistants, reinforcers, outsiders, and defenders shape social rewards (Salmivalli et al., 1996). The framework therefore uses “socioecological” consistently and organizes prevention and recovery at multiple levels.

The best available syntheses and evidence-based guidance indicate modest, heterogeneous effects (Community Preventive Services Task Force, 2022). Gaffney et al. (2021) reported random-effects estimates favoring school-based programs for perpetration (OR 1.309, 95% CI 1.24–1.38; I^2^ = 73.7%) and victimization (OR 1.244, 95% CI 1.19–1.31; I^2^ = 77.5%). These odds ratios summarize relative effects on the odds scale and should not be interpreted as absolute percentage-point reductions. In 69 randomized trials, Fraguas et al. (2021) found a small improvement in a broad mental health-problems category at the end of intervention (Cohen d = −0.205, 95% CI −0.277 to −0.133; k = 20; I^2^ = 83.7%). Dependence among outcomes, high heterogeneity, and possible publication-bias sensitivity mean that this estimate should not be treated as a universal program effect.

Crucially, incident reduction and mental health recovery are not equivalent, but neither synthesis alone proves that claim. Guzman-Holst et al. (2022) pooled 22 controlled studies and found very small post-intervention effects on internalizing symptoms (Hedges g = 0.06, 95% CI 0.028–0.101), anxiety (g = 0.08, 0.011–0.158), and depression (g = 0.06, 0.014–0.107) (Guzman-Holst et al., 2022). The difference from Fraguas et al. likely reflects eligibility (randomized-only versus controlled designs), outcome breadth (“mental health problems” versus internalizing domains), extraction and dependence rules, and different standardized metrics. The estimates are not directly comparable. Together, they support a cautious conclusion: universal anti-bullying programs can confer population benefit, but their average mental health effects are small and do not establish recovery for students with clinically significant sequelae.

Cyberbullying programs also yield modest effects. Meta-analyses support reductions in cyberbullying perpetration and victimization, but outcomes vary and many programs focus on knowledge or attitudes rather than mental health (Gaffney et al., 2019; Polanin et al., 2022). Effective digital prevention should address privacy, consent, bystander behavior, moral disengagement, platform reporting, evidence preservation, group-chat dynamics, and continuity between online and school settings. Restrictive monitoring alone can drive behavior into less visible channels and deter disclosure.

Programs such as KiVa illustrate the value of changing peer-group norms and reinforcing defending behavior; evaluations have found reductions in victimization and improvements in anxiety, depression, or perceptions of peers, generally with small effect sizes (Williford et al., 2012). However, no program is universally effective. The healthy context paradox warns that remaining victims may require more—not less—support as overall prevalence declines (Huitsing et al., 2019). Universal prevention therefore needs a selective and indicated care pathway.

The two-track model aligns with multi-tiered systems of support: universal prevention and climate work correspond to Tier 1; rapid, selective support for exposed or at-risk students to Tier 2; and indicated mental health and safeguarding intervention to Tier 3 (McIntosh & Goodman, 2016). The proposed contribution is not a new tier structure. It is the explicit linkage of exposure control, candidate mechanisms, role-specific action, and recovery outcomes across tiers. The complete package remains a proposed synthesis framework requiring prospective evaluation.

In this review, “trauma-informed” describes an interpretive and ethical lens—safety, trustworthiness, choice, collaboration, cultural responsiveness, and avoidance of re-traumatization—not a claim that a complete trauma-informed school model has proven efficacy (Substance Abuse and Mental Health Services Administration [SAMHSA], 2014). A 2019 systematic review found no eligible rigorous evaluations of whole-school trauma-informed approaches (Maynard et al., 2019), although school-based mental health prevention has a broader evidence base (Feiss et al., 2019). Accordingly, trauma-informed principles are classified as evidence-informed practice guidance, and any named program still requires evaluation of symptoms, violence, attendance, equity, implementation, and unintended effects.

## 4. Discussion

### 4.1. The Central Implication: Exposure Reduction and Recovery Must Be Co-Designed

The synthesis supports a cautious practical conclusion: reducing average exposure and promoting recovery among already affected students are related but non-equivalent objectives. Whole-school prevention changes exposure probability and peer norms; selective and indicated care addresses continuing symptoms, mechanisms, and functioning. This distinction integrates established prevention and school mental health principles rather than claiming that either component is individually novel.

This distinction also improves evaluation. A program may reduce perpetration without improving victimization, improve reporting while temporarily increasing recorded incidents, or improve school climate while leaving severe victims isolated. Mental health outcomes may lag behind exposure reduction, and symptom improvement may occur through enhanced support even when incident counts change little. Trials and quality-improvement systems should therefore specify causal pathways and measure exposure, mediator, symptom, functioning, and implementation outcomes at multiple time points.

Figure 1 is a hypothesis-generating logic model. Direct evidence is strongest for distrust and belonging as mediators and for a rumination-sleep temporal sequence; mappings for threat, humiliation/shame, and social defeat are conceptually grounded but require prospective mediation and intervention tests. The framework should improve evaluation by requiring programs to specify which exposure, appraisal, mechanism, symptom, and implementation outcome is expected to change, on what timescale, and for whom.

### 4.2. A Clinically Safe Response Pathway

Every report indicating possible bullying or violence should prompt two proportionate questions: Is the young person safe now? and Has the reported exposure affected mental health or functioning? Immediate triage should consider ongoing access by the alleged perpetrator, threats, sexual content, weapons, doxxing, non-consensual imagery, retaliation, family or community violence, and safeguarding obligations. The response should preserve confidentiality as far as legally and practically possible, explain limits of confidentiality before sensitive disclosure, and avoid repeated retelling to multiple adults.

Psychosocial assessment should be proportionate to severity, persistence, developmental context, and functional impact, while avoiding both minimization and unnecessary pathologization. Evidence-supported screening instruments may assist trained professionals, but positive screens are not diagnoses. Questions should cover mood, anxiety, trauma symptoms, sleep, somatic distress, school avoidance, concentration, self-harm, suicidal thoughts or behavior, substance use, aggression, and support. Current or imminent self-harm or suicide risk, an immediate serious threat, or weapon involvement requires urgent action under local safeguarding and emergency procedures. Other disclosures of self-harm or sexual violence require timely, trauma-informed safeguarding and clinical assessment according to local law and policy.

School nurses and other health professionals occupy a strategically important position because students may present with headaches, abdominal pain, fatigue, sleep problems, injuries, medication concerns, or recurrent requests to leave class rather than directly naming bullying. A trauma-informed encounter validates the experience, assesses safety, documents objectively, avoids implying blame, coordinates with designated school personnel, and arranges follow-up. Follow-up is essential because symptoms and retaliation may emerge after the initial report and because a closed disciplinary case does not demonstrate psychological recovery.

Restorative practices, peer mediation, or joint meetings may be considered only when participation is voluntary, safety is credible, and power asymmetry has been addressed. Excluding forced mediation in coercive, identity-based, sexual, or severe violence is presented here as an author-generated safeguarding recommendation rather than a demonstrated mechanism-specific treatment effect. Local legal, child-protection, and professional standards take precedence. Accountability should be transparent and proportionate, while educational or therapeutic support is offered to perpetrators according to assessed need.

### 4.3. Implementation Principles for Schools and Systems

First, definitions and reporting categories should be behaviorally specific. Students and staff need to know the difference between conflict, aggression, bullying, harassment, discrimination, sexual violence, and credible threats because response thresholds differ. Repetition should not become a reason to delay action when a single severe digital or physical incident creates continuing harm.

Second, reporting systems must be trusted. Multiple routes—named, confidential, and where feasible anonymous—should be accessible to students with disabilities and language differences. Schools should publish response steps, protect reporters from retaliation, communicate what can and cannot remain confidential, and provide feedback without disclosing another student’s private information. Perceived inaction is itself a mechanism of institutional betrayal and future non-disclosure.

Third, schools should monitor implementation and equity. Useful indicators include incident prevalence, location and modality, time to first protective action, recurrence, student-perceived safety, belonging, disclosure confidence, absenteeism, symptom screening where ethically appropriate, referral completion, and disciplinary disproportionality. An increase in reports after launching a trusted system can indicate improved detection rather than worsening violence; interpretation requires triangulation.

Fourth, student voice should shape design without making students responsible for enforcement. Youth can identify digital practices, hidden locations, barriers to disclosure, stigmatizing policy language, and feasible peer-support strategies that adults miss. Participation should include students from marginalized and disabled groups and must not expose individual victims.

Fifth, fidelity and adaptation must be balanced. Core functions—clear norms, adult responsiveness, supervision, peer reinforcement change, parent communication, targeted case management, and data feedback—should be preserved, while language, examples, digital platforms, reporting routes, and referral pathways are culturally adapted. Transferability is not guaranteed: legal definitions, class size, staff capacity, collectivist or individualist norms, digital access, disability inclusion, and mental health resources differ across settings. Resource-constrained systems may prioritize trusted reporting, rapid protection, trained adult response, and referral continuity before adopting complex branded programs. Recommendations concerning U.S. school shootings and drills should remain explicitly U.S.-bounded.

### 4.4. Research Priorities

**Mechanism-focused causal designs:** Future studies should move beyond cross-sectional mediation. Repeated-measures cohorts, intensive longitudinal designs, natural experiments, sibling/twin comparisons, and intervention-mediated analyses can test whether changes in distrust, shame, rumination, sleep, and belonging precede changes in symptoms. Time-varying exposure and recovery should be modeled explicitly.

**Role and trajectory precision:** Binary ever/never measures conceal chronic, intermittent, escalating, and remitting trajectories. Research should distinguish victim, perpetrator, perpetrator–victim, defender, reinforcer, outsider, and witness roles across online and offline settings and examine transitions between them. Person-centered models should be validated across cultures rather than treated as universally equivalent.

**Outcome completeness:** Prevention trials should measure not only bullying frequency but also depression, anxiety, trauma symptoms, sleep, loneliness, self-harm, suicidality, externalizing behavior, substance use, school belonging, attendance, academic function, perceived safety, and adverse effects. Serious outcomes require adequately powered designs, standardized definitions, and ethical crisis protocols.

**Equity and context:** Low- and middle-income countries, conflict-affected settings, migrant and refugee youth, disabled students, and intersectional identities remain underrepresented. Global research should avoid applying high-income school assumptions to settings with different class sizes, digital access, legal structures, or mental health resources. Bias-based motives and structural discrimination should be measured directly.

**Digital specificity:** Platform affordances change faster than many research cycles. Studies should measure audience size, anonymity, persistence, algorithmic amplification, generative or manipulated media, group-chat exclusion, gaming contexts, reporting outcomes, and offline-online continuity. Platform interventions require independent evaluation, transparent safety metrics, and attention to displacement into other channels.

**Implementation and economic evaluation:** Comparative effectiveness studies should identify which program functions work, for whom, at what cost, and under what staffing conditions. Hybrid effectiveness-implementation trials can assess reach, fidelity, adaptation, sustainability, and equity. Cost analyses should include avoided mental health treatment, absenteeism, educational loss, staff time, and long-term social consequences.

**Recovery and iatrogenic effects:** Few studies test whether targeted psychological care after victimization improves recovery or which treatments fit particular mechanisms. Research should also monitor unintended effects, including retaliation, stigma after disclosure, over-surveillance, exclusionary discipline, forced mediation, or intensified isolation of remaining victims in low-bullying contexts.

### 4.5. Strengths and Limitations of This Review

The review’s principal strength is its explicit integration of epidemiology, developmental psychopathology, school organization, clinical response, and intervention science. The evidentiary hierarchy is reported transparently: uncertainty and heterogeneity accompany quantitative anchors, within-person inference is distinguished from between-person association, and the proposed framework is identified as evidence-informed and hypothesis-generating. Supplementary File S1 documents the purposive search and synthesis procedure, while Supplementary Table S1 summarizes the characteristics, contributions, and limitations of the principal evidence sources.

Limitations remain. The search was structured and documented but purposive, not exhaustive; no prospectively maintained screening log or formal study-level risk-of-bias assessment was completed, and a reliable denominator for all examined records cannot be reconstructed. English-language evidence and high-income settings predominate. Many studies rely on self-report, cross-sectional designs, non-invariant measures, and heterogeneous definitions, lags, and outcomes. The framework combines pathways with different evidence status and has not been prospectively validated. School-shooting and drill evidence is particularly U.S.-specific; trauma-informed whole-school models lack rigorous effectiveness evidence; and some practice mappings are expert recommendations. Publication bias, residual confounding, common-method variance, and rapid change in digital platforms further limit universal inference.

## 5. Conclusions

Bullying and school violence are preventable exposures and potentially modifiable risk factors for adverse youth mental health outcomes. Robust associations span depression, anxiety, trauma symptoms, sleep disturbance, loneliness, self-harm, suicidality, substance use, and externalizing problems, but magnitude and certainty vary by design and measurement. Prospective, twin, and quasi-experimental evidence supports a contributory role for bullying in at least some short- and medium-term outcomes while leaving reciprocal vulnerability, social selection, and residual confounding important.

The central integrative conclusion is that fewer incidents do not necessarily demonstrate psychological recovery. Whole-school prevention remains foundational, but systems should also provide rapid protection, proportionate confidential assessment, targeted care, supported re-entry, and follow-up. The proposed mechanism-to-action framework links exposure prevention with measurement of distrust, rumination, sleep, belonging, symptoms, and functioning; threat appraisal, humiliation/shame, and social defeat remain hypotheses requiring stronger tests. Effectiveness of the full two-track architecture has yet to be evaluated. Progress should be judged by safety, belonging, equity, functioning, and recovery, not disciplinary counts alone.

## Figures and Tables

**Table 1:** Operational boundaries used in the narrative synthesis.

Construct	Defining Features	Examples	Mental Health Relevance
**Bullying**	Intentional aggression; repetition or high likelihood of repetition; power imbalance.	Physical, verbal, relational, social exclusion, property damage.	Chronic interpersonal threat and constrained capacity to escape.
**Cyberbullying**	Digitally mediated aggression with power shaped by anonymity, technological skill, audience, permanence, and replicability.	Harassing messages, rumor spreading, impersonation, exclusion, non-consensual sharing.	Potentially continuous exposure, uncontrollable audience, persistent or resurfacing material.
**School violence**	Broader set of harmful acts occurring in school-related contexts; repetition and power imbalance are not required.	Physical attacks, fighting, sexual harassment/coercion, threats, weapon incidents, staff violence.	Acute trauma, chronic fear, injury, disrupted safety, avoidance, and institutional distrust.
**Polyvictimization**	Exposure to multiple forms, perpetrators, settings, or periods of victimization.	Offline bullying plus cyberbullying; school violence plus family/community violence.	Cumulative load, fewer safe contexts, greater symptom severity and complexity.
**Role involvement**	Victim, perpetrator, perpetrator–victim, defender, reinforcer, outsider, or witness; roles may change over time.	A student may be targeted at school, retaliate online, and witness violence elsewhere.	Different pathways to internalizing, externalizing, guilt, fear, and suicidality.
**Peer victimization**	Harmful treatment by peers; may include isolated or non-power-imbalanced aggression and therefore is not synonymous with bullying.	Peer aggression, exclusion, harassment, coercion.	Broader exposure category useful when studies do not measure repetition and power imbalance.

**Table 2:** Quantitative evidence anchors, uncertainty, and principal evidence-certainty limitations. Metrics are not directly comparable across rows; no conversion to a common effect scale was attempted because exposures, outcomes, designs, and estimands differ.

Evidence Source	Design/Sample	Estimate (95% CI; Heterogeneity)	Interpretation	Principal Certainty Limitation
UNESCO (2019)	Surveillance; 144 countries/territories	32% bullied in previous month	Burden estimate; not comparable across instruments	Coverage, wording, reporting norms
WHO/HBSC (Cosma et al., 2024)	~280,000 adolescents; 44 countries/regions	11% school-bullied; 15% cyberbullied	Instrument-specific prevalence	Regional rather than global; self-report
Ye et al. (2023)	31 cross-sectional studies; *n* = 133,688	Victim OR 2.77 (2.29–3.35); perpetrator 1.73 (1.34–2.23); perpetrator–victim 3.19 (2.54–4.01); I^2^ > 50%	Association; ORs are not risk ratios	Cross-sectional; residual confounding; publication bias
van Geel et al. (2014)	34 ideation studies (*n* = 284,375); 9 attempt studies (*n* = 70,102)	Ideation OR 2.23 (2.10–2.37), I^2^ = 85.2%; attempts OR 2.55 (1.95–3.34), I^2^ = 88.9%	Association, not individual causation	High heterogeneity; mostly self-report/cross-sectional
Lee et al. (2026)	27 longitudinal studies; *n* = 13,497	Pooled prospective r = 0.23	Small pooled zero-order temporal association; not a within-person effect	English-only; outcome mix; baseline adjustment varied
Singham et al. (2017)	Twin-difference cohort; *n* = 11,108	Most adjusted effects attenuated by 5 years; paranoid thoughts and cognitive disorganization persisted	Strengthens control of familial/genetic confounding	Attrition; residual nonshared confounding
Gaffney et al. (2021)	100 evaluations; 103 effects	Perpetration OR 1.309 (1.24–1.38), I^2^ = 73.7%; victimization OR 1.244 (1.19–1.31), I^2^ = 77.5%	Intervention-favoring ORs; not absolute percentage-point reductions	Substantial heterogeneity; mixed randomized/nonrandomized designs
Fraguas et al. (2021)	69 RCTs; 77 samples; *n* = 111,659	Mental health problems Cohen d = −0.205 (−0.277 to −0.133), k = 20, I^2^ = 83.7%	Small effect across broad mental health category	Outcome dependence/breadth; high heterogeneity; publication-bias concerns
Guzman-Holst et al. (2022)	22 controlled studies; *n* = 58,091	Internalizing Hedges g = 0.06 (0.028–0.101); anxiety 0.08 (0.011–0.158); depression 0.06 (0.014–0.107)	Very small domain-specific effects	Different eligibility/outcome rules; mixed designs
Rossin-Slater et al. (2020)	Quasi-experiment; 44 fatal U.S. shootings	21.4% relative increase in local youth antidepressant use over 2 years	Context-specific proxy outcome	U.S.-specific; prescriptions are an indirect outcome

**Table 3:** Mechanism-to-action matrix for prevention and response.

Mechanism/Evidence Status	Observable Indicators	Response and Primary Actor	Evaluation Target/Example Measure	Recommendation Status
Threat/hypervigilance—theory-supported candidate	Scanning, startle, avoidance, somatic complaints	Immediate safety plan and predictable contact—school safeguarding + school health	Perceived safety; CATS trauma symptoms (Sachser et al., 2017); validated anxiety measure	Evidence-informed; mediator effect untested
Humiliation/shame—theory-supported candidate	Self-blame, secrecy, withdrawal, identity concerns	Validate harm; preserve dignity/confidentiality; identity-affirming support—school health/mental health	Shame scale; self-esteem; disclosure safety	Author-proposed mapping
Interpersonal distrust/social defeat—prospective mediation for distrust	Expecting betrayal; avoidance; hostile interpretations	Reliable follow-through; safe peer reconnection—education + mental health	Interpersonal distrust (Tsomokos & Slavich, 2024); help-seeking; loneliness	Empirical mediator + evidence-informed action
Rumination/sleep disruption—longitudinal temporal sequence	Replaying events, late checking, insomnia, fatigue	Reduce re-exposure; coping and sleep intervention—family + mental health	Age-appropriate rumination measure; PSQI/ASWS after local validation; daytime function	Temporal evidence; mediated intervention untested
School disconnection—longitudinal mediation	Absence, poor concentration, declining engagement	Named advocate; supported re-entry; participation plan—education	PSSM belonging (Goodenow, 1993); attendance; engagement	Empirical mediator + evidence-informed action
Aggression/retaliation—associated pathway	Fighting, online retaliation, weapon concern	Functional assessment; proportionate accountability; threat assessment if indicated—school + specialist services	SDQ externalizing (Goodman, 1997); recurrence; disciplinary equity	Evidence-informed; mechanism-specific efficacy uncertain

**Table 4:** Proposed two-track, multilevel prevention and recovery architecture. This synthesis is compatible with multi-tiered systems of support but has not been prospectively evaluated as an integrated package.

Level/Primary Actor	Track A: Prevent and Stop Exposure	Track B: Detect and Treat Harm	Minimum Indicators	Evidence Status
System/policy	Clear definitions; rights-based policy; safe reporting; platform and law-enforcement protocols; equitable resources.	Referral standards; crisis pathways; continuity across health, education, and child protection.	Prevalence, response time, referral completion, equity, recurrence.	Policy/implementation synthesis
School	Whole-school program; staff training; hot-spot supervision; student voice; confidential reporting; implementation team.	Proportionate psychosocial triage after reports indicating possible harm, without waiting for disciplinary substantiation; named case coordinator; follow-up.	Safety, belonging, disclosure trust, symptoms, attendance, fidelity.	Program and practice evidence
Classroom/peer	Norm-setting; cooperative learning; digital citizenship; safe defending; rapid interruption.	Reintegration and peer support; monitor isolation; avoid forced mediation or public disclosure.	Peer norms, defending, classroom climate, social isolation.	Evidence-informed school practice
Family	Warm monitoring; non-punitive digital discussion; coordinated plan; avoid retaliation.	Observe sleep, mood, avoidance, self-harm, substance use; support treatment and school re-entry.	Communication, adherence, recurrence, family strain.	Evidence-informed family guidance
Health professionals	Ask about school and online safety during age-appropriate assessment; document and safeguard.	Assess depression, anxiety, trauma, sleep, self-harm, suicide risk, substance use, and functioning; safety plan and refer.	Symptom trajectory, functioning, safety-plan use, care access.	Clinical/safeguarding guidance
Digital platforms	Friction for forwarding; privacy-by-default for minors; rapid reporting; account and content safeguards.	Preserve evidence; reduce re-exposure; provide age-appropriate crisis and support pathways.	Removal latency, repeat targeting, appeal outcomes, user safety.	Author-proposed platform principles

## Data Availability

No primary data were generated for this narrative review. The evidence sources are cited in the article and summarized in Supplementary Table S1; therefore, no additional dataset is available.

## Supplementary Materials

The following supporting information can be downloaded at https://www.mdpi.com/article/10.3390/bs16101815/s1.
