# Supplementary material for: From Exposure to Psychological Harm: A Socioecological and Trauma-Informed Narrative Review of Bullying, School Violence, and Youth Mental Health
Source: Behav Sci (Basel). 2026 Oct 4;16(10):1815. doi: 10.3390/bs16101815 (PMC13701556; doi:10.3390/bs16101815)
Supplement: Supplementary file 1 [file behavsci-16-01815-s001.zip › behavsci-4559602-supplementary File S1.pdf]

Supplementary File S1

From Exposure to Psychological Harm: A Socioecological and Trauma-Informed Narrative Review of Bullying, School Violence, and Youth Mental Health

Search Strategy and Evidence-Selection Audit Trail

The review used a structured, purposive narrative-review search rather than exhaustive systematic-review screening. The original submission on 29 July 2026 was based on literature identified before submission. During revision, both authors reran the structured searches in full from 15 to 27 August 2026 and completed a final update on 8 September 2026. Reporting was guided by SANRA. Because no prospectively maintained screening log was used, a PRISMA denominator cannot be reconstructed and no record-flow count is claimed.

S1.1. Concept blocks

Block	Terms
E: exposure	bully* OR "peer victimization" OR cyberbully* OR "school violence" OR "school shooting*" OR "school harassment" OR "school aggression"
P: population	child* OR adolescen* OR youth* OR student* OR "school-aged"
O: outcomes	"mental health" OR depress* OR anxi* OR trauma* OR PTSD OR suicid* OR "self-harm" OR sleep OR loneliness OR internalizing OR externalizing OR substance* OR "school belonging" OR attendance
M: mechanisms/modifiers	distrust OR rumination OR shame OR humiliation OR "social defeat" OR hypervigilance OR belonging OR mediator* OR moderator* OR resilience OR "social support"
I: intervention/implementation	intervention* OR prevent* OR program* OR "whole-school" OR "trauma-informed" OR screening OR treatment OR implementation OR evaluation

S1.2. Database-specific strategies

The principal logic was E AND P AND (O OR M OR I). Focused searches paired E and P separately with mechanism, moderator, measurement, causal-inference, intervention, and implementation terms to reduce the risk that a single broad string would miss narrowly indexed studies. Syntax below documents the representative full strings and fields used during the revision rerun; spelling variants were adapted to each platform.

Source	Fields/coverage	Representative strategy	Limits and role
PubMed/MEDLINE	Title/Abstract plus MeSH where available	(("Bullying"[Mesh] OR bully*[tiab] OR "peer victimization"[tiab] OR cyberbully*[tiab] OR "school violence"[tiab] OR "school shooting*"[tiab]) AND (child*[tiab] OR adolescen*[tiab] OR youth*[tiab] OR student*[tiab]) AND (("Mental Health"[Mesh] OR depress*[tiab] OR anxi*[tiab] OR trauma*[tiab] OR suicid*[tiab] OR "self-harm"[tiab] OR sleep[tiab] OR loneliness[tiab]) OR (distrust[tiab] OR rumination[tiab] OR belonging[tiab] OR mediator*[tiab]) OR (intervention*[tiab] OR prevent*[tiab] OR program*[tiab])))	English; 2000-8 Sep 2026; no electronic age filter
APA PsycINFO (EBSCOhost)	TI/AB with subject headings expanded during focused searches	TI (bully* OR "peer victimization" OR cyberbully* OR "school violence" OR "school shooting*") OR AB (bully* OR "peer victimization" OR cyberbully* OR "school violence" OR "school shooting*") AND TI/AB	Peer reviewed where indexed; English; 2000-8 Sep 2026

		(child* OR adolescen* OR youth* OR student*) AND TI/AB ("mental health" OR depress* OR anxi* OR trauma* OR suicid* OR "self-harm" OR sleep OR loneliness OR belonging OR rumination OR distrust OR intervention* OR prevent*)	
ERIC (EBSCOhost)	TI/AB; education descriptors used for focused searches	TI/AB (bully* OR "peer victimization" OR cyberbully* OR "school violence" OR "school safety") AND TI/AB (child* OR adolescen* OR youth* OR student* OR school*) AND TI/AB ("mental health" OR well-being OR belonging OR climate OR attendance OR intervention* OR prevent* OR implementation)	English; 2000-8 Sep 2026
Scopus	TITLE-ABS-KEY	TITLE-ABS-KEY ((bully* OR "peer victimization" OR cyberbully* OR "school violence" OR "school shooting*") AND (child* OR adolescen* OR youth* OR student*) AND ("mental health" OR depress* OR anxi* OR trauma* OR suicid* OR "self-harm" OR sleep OR loneliness OR belonging OR rumination OR distrust OR intervention* OR prevent*))	Article/review; English; 2000-8 Sep 2026
Web of Science Core Collection	Topic (TS)	TS=((bully* OR "peer victimization" OR cyberbully* OR "school violence" OR "school shooting*") AND (child* OR adolescen* OR youth* OR student*) AND ("mental health" OR depress* OR anxi* OR trauma* OR suicid* OR "self-harm" OR sleep OR loneliness OR belonging OR rumination OR distrust OR intervention* OR prevent*))	Article/review; English; 2000-8 Sep 2026
Google Scholar	Title/full text; citation discovery	Representative focused strings: "bullying mental health adolescents longitudinal meta-analysis"; "cyberbullying rumination sleep longitudinal"; "school violence trauma youth systematic review"; "anti-bullying intervention internalizing meta-analysis"; "bullying school belonging mediation"	Used for forward/backward citation discovery and verification, not prevalence estimation
Publisher platforms	Targeted title/DOI/full-text verification	ScienceDirect, SpringerLink, Wiley Online Library, Taylor & Francis Online, SAGE Journals, and JAMA Network were searched by exact title, author-year combination, DOI, and focused keyword combinations.	Used to retrieve or verify candidate sources; not treated as independent exhaustive databases
Organizational sources	Site/report search	Site-specific combinations of bullying, school violence, cyberbullying, adolescent, mental health, surveillance, and intervention were used for UNESCO, WHO/HBSC, CDC/YRBS, and the Community Preventive Services Task Force.	Authoritative surveillance, policy, and evidence-guidance sources

S1.3. Eligibility and prioritization

- Population: school-aged children or adolescents; adult-only, university-only, workplace, sibling, dating-partner, and unrelated family/community violence studies were excluded unless uniquely relevant to theory or mechanisms.
- Exposure: bullying, peer victimization, cyberbullying, or violence embedded in school-related social systems.
- Evidence priority: systematic reviews/meta-analyses, representative surveillance, prospective cohorts, within-family or genetically informed studies, quasi-experiments, randomized trials, and direct mediator/moderator tests.
- Selection principles: recency, methodological rigor, sample breadth, directness, precision, and replication across contexts. Backward and forward citation chaining supplemented database retrieval.
- Extraction fields: design, population, setting, exposure, outcome, effect estimate, uncertainty, temporality, confounding control, principal limitation, and relevance to the framework.
- Verification: both authors independently checked eligibility and extraction for quantitative anchor studies and resolved discrepancies by consensus; other retained sources were checked by the second author for directness and interpretive accuracy.
- Synthesis categories: (i) empirically demonstrated associations or intervention effects; (ii) evidence-informed implications supported indirectly or across adjacent literatures; and (iii) author-proposed hypotheses or practice recommendations.
