# Supplementary material for: From Exposure to Psychological Harm: A Socioecological and Trauma-Informed Narrative Review of Bullying, School Violence, and Youth Mental Health
Source: Behav Sci (Basel). 2026 Oct 4;16(10):1815. doi: 10.3390/bs16101815 (PMC13701556; doi:10.3390/bs16101815)
Supplement: Supplementary file 1 [file behavsci-16-01815-s001.zip › behavsci-4559602-supplementary Table S1.pdf]

Supplementary Table S1

From Exposure to Psychological Harm: A Socioecological and Trauma-Informed Narrative Review of Bullying, School Violence, and Youth Mental Health

Principal Evidence Sources, Contributions, and Limitations

This table provides an audit trail for the principal empirical and synthesis sources that carry the central quantitative, causal, mechanism, vulnerability, school-violence, and intervention claims. It is not a risk-of-bias table and should not be interpreted as an exhaustive inventory of every source cited in the narrative review.

Source	Review domain	Design/sample	Principal contribution	Evidence status	Principal limitation
UNESCO (2019)	Global burden	International surveillance synthesis; 144 countries/territories	Approximately 32% bullied in previous month; broad school-violence burden	Descriptive surveillance	Definition, coverage, and reporting norms vary
Cosma et al. (2024)	School/cyberbullying prevalence	HBSC report; ~280,000 adolescents; 44 countries/regions	11% school-bullying victimization; 15% cybervictimization	Cross-national surveillance	Regional coverage and self-report; instrument-specific estimates
Young et al. (2024)	U.S. surveillance	2023 YRBS cross-sectional surveillance	Bullying clusters with sadness/hopelessness and suicide risk	Association	Cross-sectional; U.S. high-school students
Vivolo-Kantor et al. (2014)	Measurement	Systematic review/content analysis	Substantial variation in bullying and cyberbullying measures	Measurement synthesis	Heterogeneity limits prevalence comparability
Ye et al. (2023)	Depressive symptoms	Meta-analysis; 31 cross-sectional studies; n=133,688	Victims OR 2.77; perpetrators OR 1.73; perpetrator-victims OR 3.19	Pooled association	Cross-sectional; high heterogeneity; publication bias
van Geel et al. (2014)	Suicidality	Meta-analysis; 34 ideation and 9 attempt studies	Victimization associated with ideation (OR 2.23) and attempts (OR 2.55)	Pooled association	High heterogeneity; predominantly cross-sectional self-report
Christina et al. (2021)	Directionality	Systematic review/meta-analysis of longitudinal studies	Supports bidirectional between-person associations	Longitudinal association	Conventional cross-lagged estimates do not prove within-person feedback
Reijntjes et al. (2010)	Directionality	Longitudinal meta-analysis	Peer victimization and internalizing problems predict one another over time	Longitudinal association	Between-person and within-person effects not fully separated
Silberg et al. (2016)	Causal inference	Discordant monozygotic-twin study	Supports direct contributions to selected psychiatric outcomes	Genetically informed evidence	Concurrent/shorter-term outcomes; residual nonshared confounding
Singham et al. (2017)	Causal inference/recovery	Twin-difference cohort; n=11,108	Most adjusted effects attenuated by five years; selected outcomes persisted	Genetically informed longitudinal evidence	Attrition and residual nonshared confounding
Schoeler et al. (2018)	Causal inference	Meta-analysis of quasi-experimental studies	Adverse effects persist but are smaller than conventional observational estimates	Quasi-experimental synthesis	Designs and estimands remain heterogeneous
Copeland et al. (2013)	Long-term outcomes	Prospective population cohort	Childhood bullying roles associated with adult psychiatric outcomes	Longitudinal evidence	Residual confounding and context-specific cohort
Lee et al. (2026)	Cyberbullying	Meta-analysis; 27 longitudinal studies; n=13,497	Pooled prospective association r=0.23	Longitudinal pooled association	Zero-order association; heterogeneous lags and adjustments; not within-person causality
John et al. (2018)	Cyberbullying/self-harm	Systematic review	Cybervictimization associated with self-harm and suicidal behaviors	Evidence synthesis	Predominantly observational evidence
Kowalski et al. (2014)	Cyberbullying outcomes	Critical review/meta-analysis	Cyberbullying involvement associated with psychological and behavioral outcomes	Pooled association	Construct and design heterogeneity
Jose & Vierling (2018)	Rumination/sleep	Longitudinal adolescent study	Cybervictimization predicted rumination, followed by poorer sleep	Temporal sequence	Single-study pathway; not an intervention-mediated effect
Tsomokos & Slavich (2024)	Interpersonal distrust	Prospective Millennium Cohort analysis; n~10,000	Distrust partially mediated bullying-to-later mental-health associations	Prospective mediation	Does not validate the complete proposed framework
Arslan & Allen (2021)	School belonging	Longitudinal adolescent study	Belonging mediated links with emotional problems and well-being	Longitudinal mediation	Context-specific self-report and observational design
Huitsing et al. (2019)	Healthy-context paradox	Intervention-based longitudinal analysis	Some remaining victims show poorer adjustment as group victimization declines	Contextual/moderation evidence	Within-person and stable-difference explanations remain difficult to separate
Abregú-Crespo et al. (2024)	Neurodevelopmental/psychiatric vulnerability	Systematic review/meta-analysis	Higher odds of victimization and perpetrator-victim involvement	Risk-factor synthesis	Diagnostic and measurement heterogeneity
Gower et al. (2018)	LGBTQ+ youth	Critical review	Identity-based victimization is linked to substantial mental-health burden	Evidence synthesis	Variation in identity measurement and study design
Williams et al. (2021)	LGBTQ+ victimization/self-harm	Systematic review/meta-analysis	High victimization prevalence among LGBTQ+ youth with self-harm/suicide experiences	Pooled association	Selected high-risk population; observational evidence
Xu et al. (2020)	Racial/ethnic bullying	Review	Bias-based context changes exposure and health meaning	Evidence synthesis	Broad group categories and contextual heterogeneity
Polanin et al. (2020)	School violence	Systematic review/meta-analysis	School violence exposure is associated with multiple	Evidence synthesis	Broad, heterogeneous exposure definition

			adverse outcomes		
Rossin-Slater et al. (2020)	School shootings	Quasi-experiment; 44 fatal U.S. shootings	21.4% relative increase in local youth antidepressant use over two years	Quasi-experimental proxy outcome	U.S.-specific; prescriptions are an indirect outcome
Riehm et al. (2021)	Fear of school violence	Cross-sectional U.S. adolescent study	Concern about shootings associated with depression, anxiety, and panic symptoms	Association	Cross-sectional and U.S.-specific
Gaffney et al. (2021)	School anti-bullying programs	Systematic review/meta-analysis; 100 evaluations; 103 effects	Programs favor reduced perpetration and victimization on the odds scale	Intervention synthesis	Substantial heterogeneity; randomized and nonrandomized designs
Fraguas et al. (2021)	Anti-bullying and mental health	Meta-analysis; 69 RCTs; 77 samples; n=111,659	Small improvement in broad mental-health-problems outcome (d=-0.205)	Randomized intervention synthesis	High heterogeneity, outcome dependence, publication-bias concerns
Guzman-Holst et al. (2022)	Internalizing outcomes	Systematic review/meta-analysis; 22 controlled studies; n=58,091	Very small effects for internalizing symptoms, anxiety, and depression	Controlled intervention synthesis	Mixed designs and different eligibility/outcome rules
Polanin et al. (2022)	Cyberbullying intervention	Systematic review/meta-analysis	Programs reduce cyberbullying perpetration and victimization modestly	Intervention synthesis	Program components and outcomes are heterogeneous
Maynard et al. (2019)	Trauma-informed schools	Systematic review	No eligible rigorous evaluations of whole-school trauma-informed approaches	Evidence-gap synthesis	Absence of eligible studies is not evidence of ineffectiveness
Feiss et al. (2019)	School mental-health prevention	Systematic review/meta-analysis	School-based prevention can improve stress, anxiety, and depression outcomes	Intervention synthesis	Programs, populations, and outcomes vary
Marx & Kettrey (2016)	Affirming student groups	Systematic review/meta-analysis	Gay-straight alliances associated with lower LGBTQ+ victimization	Mostly observational synthesis	Selection/confounding limits causal inference
Midgett & Doumas (2019); Rivers et al. (2009)	Bystanders	Cross-sectional school studies	Witnessing bullying associated with anxiety, depressive symptoms, or distress	Association	Cross-sectional self-report; causal direction uncertain
